# Retraction of “lncRNA ACTA2-AS1 inhibits malignant phenotypes of gastric cancer cells”

**DOI:** 10.1515/med-2023-0892

**Published:** 2023-12-31

**Authors:** Zhiping Liu, Kaibing Hu, Xiang Wang, Youqian Zhang, Weiping Wang, Yindi Wu

**Affiliations:** Department of General Surgery, Hefei Hospital Affiliated to Medical University of Anhui, Hefei 230011, Anhui, China; Department of Pediatrics, Hefei First People’s Group Hospital, 390 Huaihe Road, Luyang District, Hefei 230000, Anhui, China

Retraction of: Liu Z, Hu K, Wang X, Zhang Y, Wang W, Wu Y. lncRNA ACTA2-AS1 inhibits malignant phenotypes of gastric cancer cells. Open Medicine 2022;17(1):266–279, DOI: 10.1515/med-2021-0406.

This article has been retracted by the publisher due to irregularities in the figures and lack of clarity regarding the origin of the images. As a result, the editors cannot guarantee the legitimacy of the presented data.

